# Confocal Microscopy Predicts the Risk of Recurrence and Malignant Transformation of Mucocutaneous Neurofibromas in NF-1: An Observational Study

**DOI:** 10.1155/2018/6938130

**Published:** 2018-09-09

**Authors:** Giuseppe Giudice, Giorgio Favia, Angela Tempesta, Luisa Limongelli, Michelangelo Vestita

**Affiliations:** ^1^Section of Plastic and Reconstructive Surgery, Department of Emergency and Organ Transplantation, University of Bari, 11, Piazza Giulio Cesare, Bari, 70124, Italy; ^2^Department of Interdisciplinary Medicine, Complex Operating Unit of Odontostomatology, “Aldo Moro” University, Piazza G. Cesare 11, 70124 Bari, Italy

## Abstract

From 2005 to 2010, 20 consecutive patients with fully manifested neurofibromatosis type 1 (NF1) underwent elective neurofibroma resection at our institution (Departments of Plastic Surgery and of Odontostomatology). Specimens were photographed under optical microscope and confocal laser scanning microscopy (CLSM) with ultra-high accuracy of detail, including depth of field. Patients were followed up for a minimum of 4 years and up to a maximum of 12 years, postsurgery. While all nonrecurring lesions showed intense fluorescence, six of the seven lesions with absence of fluorescence under CLSM recurred at a mean of 5.5 years after surgical excision. Among the re-excised lesions, 3 were diagnosed as malignant at the subsequent removal. Despite the limitation of a small cohort, CLSM appears to be a simple and low-cost technique to differentiate forms of neurofibromas with low and high risk of recurrence and malignant degeneration.

## 1. Introduction

We retrospectively describe our 5-year experience in using confocal laser scanning microscopy (CLSM) to differentiate the morphological features of Schwann cells at the time of first resection of neurofibromas arising in NF1 patients (study registration number: researchregistry3681). Based on our experience, we propose that CLSM could be a simple low-cost technique to differentiate neurofibromas with low or high risk of recurrence and malignant degeneration.

## 2. Report

From 2005 to 2010, 20 consecutive patients with fully manifested neurofibromatosis type 1 (NF1) underwent elective neurofibroma resection at our institution (Departments of Plastic Surgery and of Odontostomatology). The following demographic and clinical data were retrospectively included in our analysis: age, sex, location and type of neurofibroma excised, family history of NF1, recurrence after excision, time frame of recurrence, malignant degeneration, and other associated pathologies. All patients underwent wide (1.5 to 2 cm margins) surgical excision of burdening, symptomatic neurofibromas, with consequent direct surgical reconstruction of the defects, using local flaps or skin grafts. Excised specimens were fixed in 10% buffered formalin, paraffin-embedded, and cut in 8-12 *μ*m thick sections and stained with hematoxylin and eosin, Masson's trichrome, and picrosirius red. Histological examination was carried out using Nikon Eclipse E-600 microscope, equipped with Argon-ion and Helium-neon lasers emitting 488 and 543 nm wavelengths, which allows both optical and confocal laser scanning analyses. Specimens were photographed and images were processed using the EC-1 software, with ultra-high accuracy of detail, including depth of field.

Patient characteristics are summarized in Table 1.

**Figure 1:**
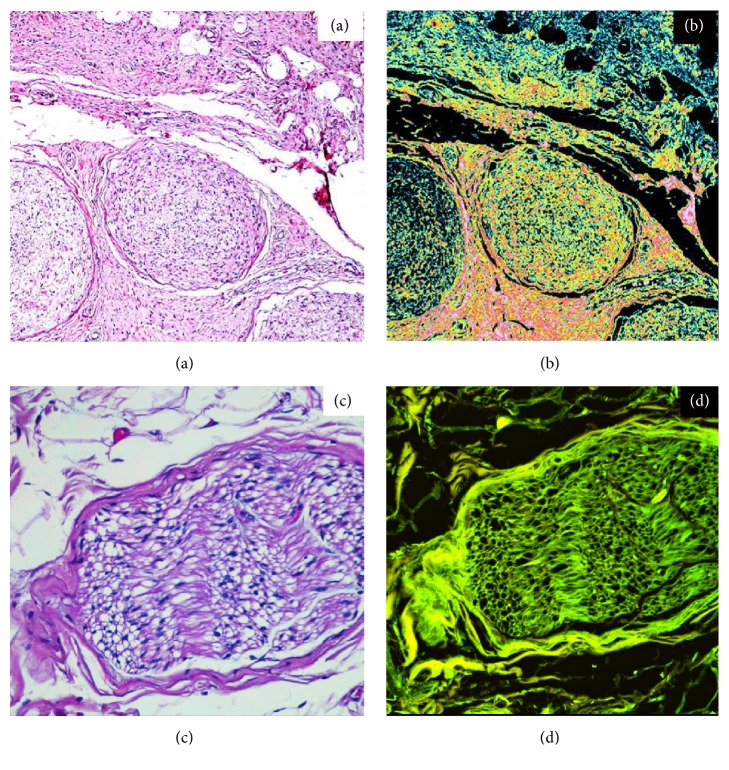
45X. Traditional optical (a) and confocal laser scanning (b) analyses showing well differentiated Schwann cells in a multinodular plexiform neurofibroma and their intense fluorescence. Fields (c) and (d) show a normal nerve as comparison, respectively, in traditional optic and confocal laser scanning. A normal nerve also shows intense fluorescence. However, several features separate a neurofibroma from a normal nerve: an increase in cell density, an increase in the number of mitosis, and an increase in the number of spindle shaped cells, nuclear anomalies, and the presence of cells of a different nature (such as macrophages, mast cells, and histiocytes).

**Figure 2:**
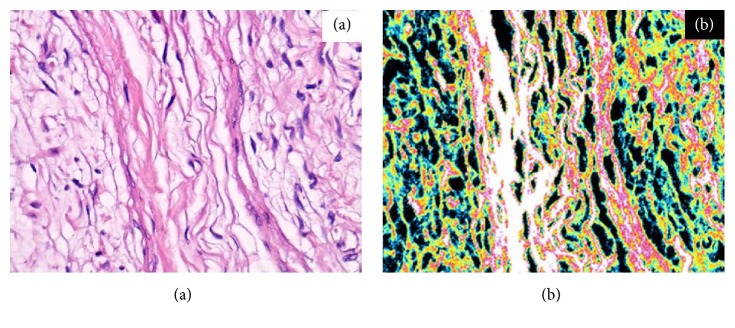
150X. Traditional optical (a) and confocal laser scanning (b) analyses of a subcutaneous/nodular neurofibroma and its intense fluorescence due to the high content of neurofibromin in the well differentiated Shwann cells.

**Figure 3:**
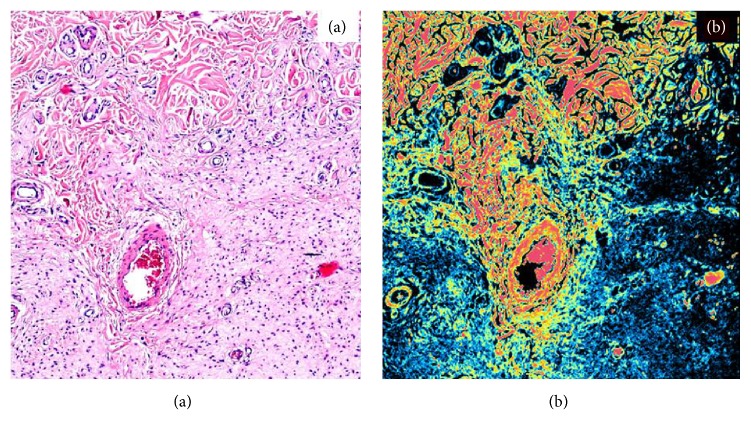
50X. Traditional (a) and confocal laser scanning (b) analyses of an infiltrating plexiform neurofibroma with lack of fluorescence of proliferating Schwann cells in opposition with the high fluorescence of the fibroblasts of the deep dermis.

Of the 20 lesions, 6 were subcutaneous/nodular, 9 were plexiform and 5 were diffuse. Schwann cells with similar morphological aspects under conventional microscopic investigation showed different patterns of endogenous autofluorescence during CLSM analyses at the time of first resection. These differences are due to their differentiation grade and to the percentage of residual neurofibromin in the cytoplasm: differentiated cells, rich in neurofibromin, showed an intense red and green fluorescence (Figures 1 and 2); less differentiated cells with minor neurofibromin content showed minimal fluorescence (Figure 3). Of the 20 lesions, 13 showed a high/medium grade of fluorescence, instead 7 showed a lack of fluorescence.

Patients were followed up for a minimum of 4 years and up to a maximum of 12 years, postsurgery. Six of the 7 lesions that showed a lack of fluorescence recurred at a mean of 5.5 years after surgical excision. The clinical-histological type of recurrent lesions was as follows: 2 subcutaneous/nodular, 2 plexiform, and 2 diffuse lesions. All recurrent lesions were subsequently re-excised with larger margins, except in case 1 (Table 1) in which partial resolution of the neurofibroma was achieved with concomitant chemotherapy for the treatment of advanced melanoma [1]. Among the re-excised lesions, 3 were diagnosed as malignant at the subsequent removal (1 nodular, 1 diffuse, and 1 subcutaneous lesions). All recurring lesions showed absence of fluorescence under CLSM, with the exception of one case showing minimal fluorescence.

## 3. Discussion

CLSM appears to be a simple and low-cost technique to differentiate morphological features of apparently identical populations of Schwann cells and, thus, to differentiate forms of neurofibromas with low and high risk of recurrence and malignant degeneration. Specifically, we observed that neurofibromas that show negative laser fluorescence have a greater tendency to develop local recurrence and a greater risk of malignant degeneration. Of note, there was no correlation between the incidence of recurrence∖malignant degeneration and the clinical∖histological type of neurofibroma in our cohort. These observations might tentatively be linked to peculiar aspects of the maturation and differentiation of Schwann cells in neurofibromas, which might translate into different concentrations of the cytoplasmatic content of neurofibromin, arising from differences in the morphological expression of genetic alterations of the NF1 gene (monoallelic or biallelic) [2–6] and the neural microenvironment [7]. Of course this is just speculation at present, and further immunohistochemical studies will need to demonstrate the possible link between neurofibromin content, fluorescence, and neurofibroma behavior. As a supplementary observation, the occurrence of a case of melanoma in our limited cohort seems to support the role NF1 and neurofibromin in neural crest-derived neoplasms, such as melanoma and other tumors [8–12]. More notable was the spontaneous partial regression of most neurofibromas (including a recurrent lesion after excision) when the patient underwent chemotherapy for his advanced melanoma [1].

In conclusion, despite the limitation of a small cohort, our preliminary data are encouraging and merit further assessment in a larger multicentre study. A correlation study to investigate the relationship between CLSM and molecular and genetic markers in Schwann cells would also be beneficial to further clarify the underlying structural and behavioral differences in these apparently indistinguishable cells.

## Figures and Tables

**Table 1:** Characteristics of the studied population and relative CLSM data.

**Age**	**Sex**	**Excised neurofibroma site**	**Excised neurofibroma histo-type**	**Oral lesions**	**NF family history **	**Comorbidities**	**Recurrence**	**Time of recurrence (years)**	**Malignant degeneration**	**Schwann cells fluorescence**
31	m	trunk	plexiform	x	x	advanced melanoma	x (regression with chemo)	6		none

47	f	lower limbs	subcutaneous/nodular							high

47	f	upper limb	diffuse	x			x	4	x	none

26	m	lower limbs	plexiform		x					high

42	f	upper limb	diffuse	x	x					high

73	f	trunk	plexiform	x		arterial hypertension, diabetes				high

44	m	trunk	Subcutaneous/nodular		x					high

16	m	oral	plexiform	x						high

59	f	upper limb	Subcutaneous/nodular	x			x	8		minimal

46	f	oral	plexiform	x						high

53	f	lower limbs	diffuse		x					high

53	f	face	plexiform			hypothyroidism				high

35	f	trunk	diffuse				x	7		none

41	m	oral	Subcutaneous/nodular	x	x					high

55	2	trunk	plexiform	x	x	arterial hypertension	x	3	x	none

50	2	face	diffuse	x						high

42	2	lower limbs	Subcutaneous/nodular		x					high

43	1	upper limb	plexiform	x						high

51	2	oral	Subcutaneous/nodular	x	x	arterial hypertension	x	5	x	none

47	2	lower limbs	plexiform		x					high

## Data Availability

The data used to support the findings of this study are available in excel format archived at our institution and can be provided if necessary. Access to discrete patients' clinical records is permitted given patient approval and in accordance to hospital regulations.

## Additional Points

(i) Confocal laser scanning microscopy might be able to differentiate the morphological features of Schwann cells in neurofibromas. Absence of fluorescence in Schwann cells was found in neurofibromas which later recur or undergo malignant degeneration. (ii) Confocal laser scanning microscopy could be used to identify neurofibromas that tend to recur or to degenerate and therefore need a more radical surgical management or closer follow-up.
